# Calcium regulation of the human mitochondrial ATP-Mg/Pi carrier SLC25A24 uses a locking pin mechanism

**DOI:** 10.1038/srep45383

**Published:** 2017-03-28

**Authors:** Steven P. D. Harborne, Martin S. King, Paul G. Crichton, Edmund R. S. Kunji

**Affiliations:** 1 Medical Research Council Mitochondrial Biology Unit, University of Cambridge, Cambridge Biomedical Campus, Wellcome Trust/MRC Building, Hills Road, Cambridge CB2 0XY, UK; 2 School of Biomedical Sciences and Astbury Centre for Structural Molecular Biology, University of Leeds, Leeds, LS2 9JT, UK; 3 Biomedical Research Centre, Norwich Medical School, University of East Anglia, Norwich Research Park, Norwich NR4 7TJ, UK

## Abstract

Mitochondrial ATP-Mg/Pi carriers import adenine nucleotides into the mitochondrial matrix and export phosphate to the cytosol. They are calcium-regulated to control the size of the matrix adenine nucleotide pool in response to cellular energetic demands. They consist of three domains: an N-terminal regulatory domain containing four calcium-binding EF-hands, a linker loop domain with an amphipathic α-helix and a C-terminal mitochondrial carrier domain for the transport of substrates. Here, we use thermostability assays to demonstrate that the carrier is regulated by calcium via a *locking pin* mechanism involving the amphipathic α-helix. When calcium levels in the intermembrane space are high, the N-terminus of the amphipathic α-helix is bound to a cleft in the regulatory domain, leading to substrate transport by the carrier domain. When calcium levels drop, the cleft closes, and the amphipathic α-helix is released to bind to the carrier domain via its C-terminus, locking the carrier in an inhibited state.

The ATP-Mg/Pi carrier (APC) is a member of the mitochondrial carrier family of transport proteins^1^,^2^,^3^. The carrier exports phosphate and imports adenine nucleotides, resulting in their net uptake into mitochondria^1^,^2^,^3^. In contrast, the related mitochondrial ADP/ATP carrier catalyzes a strict equimolar ADP/ATP exchange, leaving the total pools of adenine nucleotides unaltered^4^. APC is regulated by cytosolic calcium to control the size of the matrix adenine nucleotide pool in response to cellular energetic demands^1^,^2^,^3^, a fundamental process for cellular growth and energy metabolism^5^.

The impact of APC2 (SCaMC3/*SLC25A23*) knockouts has been studied in mouse liver^6^, mouse neuronal tissue^7^,^8^, and that of an APC3 (SCaMC2/*SLC25A25*) knockout in a mouse model^9^. These studies highlighted the requirement for adenine nucleotide movement in and out of mitochondria for respiratory function and protection against calcium-induced permeabilisation of the mitochondrial inner membrane in stress conditions^10^. In a pathophysiological role, overexpression of APC1 can assist cancer cells to evade typical cell death pathways by increasing calcium precipitation in the mitochondrial matrix, reducing calcium induced permeabilisation of the mitochondrial inner membrane^11^. Therefore, APCs play an important role in mitochondrial function under normal conditions, but also in disease.

The ATP-Mg/Pi carriers have three domains: an N-terminal regulatory domain, a linker loop domain with an amphipathic α-helix and a C-terminal mitochondrial carrier domain^12^. The N-terminal regulatory domain forms two pairs of EF-hands fused by one long central α-helix. In the calcium-bound state the amphipathic α-helix of the linker loop domain is bound to the hydrophobic cleft of EF-hands 3 and 4, analogous to calmodulin^12^,^13^. The C-terminal carrier domain is involved in substrate transport across the mitochondrial inner membrane^14^ and has the structural fold of mitochondrial carriers. It consists of three homologous sequence repeats^15^, each containing two membrane-spanning α-helices connected by a matrix α-helix^16^. Based on the identification of a conserved central substrate binding site^17^ flanked by two salt bridge networks on either side^16^,^18^, an alternating access mechanism has been proposed for substrate transport through carrier proteins^4^,^19^. The regulatory domain is located in the intermembrane space, but its position relative to the carrier domain is unknown.

The effect of calcium regulation on the transport activity of APC has been studied in isolated rat liver mitochondria^20^, isolated yeast mitochondria^3^,^21^ and more recently in liposomes with reconstituted *Arabidopsis thaliana* APC^22^,^23^. Furthermore, the effect of calcium on adenine nucleotide uptake into mitochondria of human osteosarcoma cells has been studied in which APC1 (SCaMC1/*SLC25A24*) is the major isoform^11^. However, the effects of calcium on isolated human APC1 reconstituted into liposomes has not been reported^2^.

A molecular mechanism of calcium regulation has not been established. A capping and uncapping mechanism of calcium regulation of APC has been proposed, in which the entire calcium-free regulatory domain forms a lid to block substrate transport, but this mechanism lacks molecular detail^13^. We have proposed an alternative hypothesis based on a canonical EF-hand mechanism, in which the EF-hands close in the calcium-free state and release the amphipathic α-helix from the hydrophobic cleft to bind to the carrier domain^12^.

Here, we have probed the inter-domain interactions of the protein in the calcium-bound and calcium-free states by analysing evolutionary coupling data and state-dependent changes in thermostability, allowing us to distinguish between side chain interactions important for structure and/or those involved in the regulatory mechanism, respectively. The results show that the amphipathic α-helix is bound alternately to the regulatory domain or the carrier domain depending on the calcium levels.

## Results

### Activity, orientation and calcium sensitivity of APC1 in liposomes

**Figure 1:**
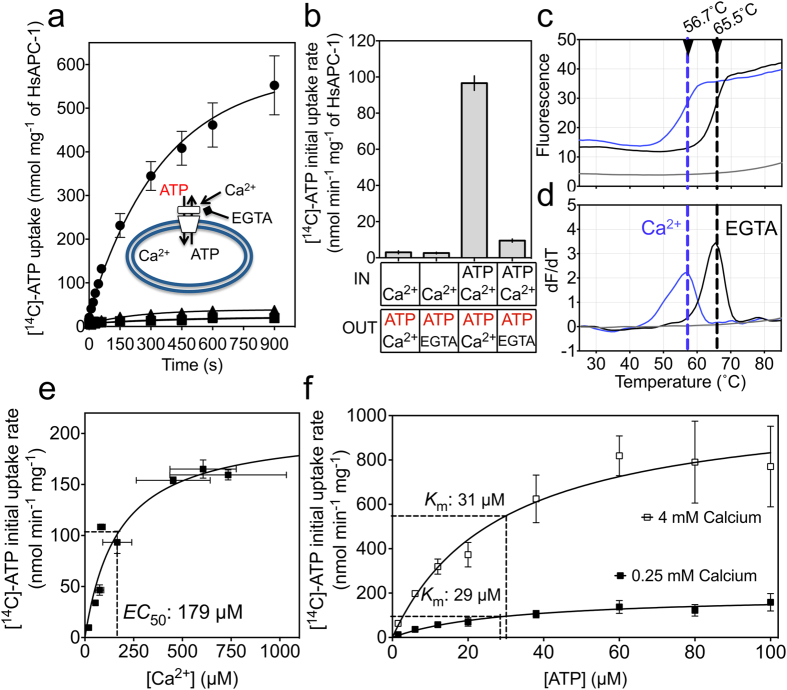
Transport activity and thermostability of wild-type APC1 in the presence or absence of calcium. (**a**) Uptake of radiolabelled ATP (red) into proteoliposomes measured with different unlabelled internal and external substrates and effectors (black). Proteoliposomes were made with 2.5 mM ATP and 1 mM calcium chloride (circles), 2.5 mM ATP and 1 mM EGTA (triangles) or no ATP and 1 mM EGTA (squares). Uptake was initiated by addition of 1.5 μM [^14^C]-ATP to the outside of liposomes. (**b**) The initial uptake rates in the conditions indicated in the table were calculated from the fit of the curves in panel *A*. (**c**) Thermostability assays with CPM. Purified carrier protein was diluted to 50 μg mL^−1^ in assay buffer, and pre-equilibrated CPM was added to 1 μg mL^−1^. Either 2.5 mM calcium chloride (blue curve) or 2.5 mM EGTA (black curve) was added prior to temperature ramping. A sample without protein was included for baseline reference (grey). **(d**) The apparent melting temperature can be derived from the inflection point of the unfolding curve from the first derivative. (**e**) APC1 proteoliposomes loaded with 2.5 mM ATP and uptake of 1.5 μM [^14^C]-ATP stimulated by the addition of 0, 0.05, 0.1, 0.5, 1, 2, 3 or 4 mM calcium chloride on the outside. Free calcium concentration was determined against a standard curve in the presence of 1 μM of fluo-5N (Supplementary Fig. 3). The relationship between initial ATP uptake rate and the calcium concentration was plotted using specific initial rates (Supplementary Fig. 3) and fitted with a curve describing hyperbolic saturation kinetics. (**f**) Uptake from APC1 proteoliposomes loaded with 2.5 mM ATP, with increasing concentrations of ATP (1.5, 6, 12, 20, 38, 60, 80 and 100 μM) added to the outside and in the presence of either 0.25 mM or 4 mM calcium chloride. The Michaelis-Menten relationship between initial ATP uptake rate (Supplementary Fig. 4) and the ATP concentration was plotted for 0.25 mM (filled squares) and 4 mM (open squares) concentrations of external calcium using the specific initial rates. Error bars represent standard deviation for measurements taken from three independent liposome preparations.

To establish an assay for calcium regulation of transport, full-length human mitochondrial ATP-Mg/Pi carrier isoform 1 (APC1) was expressed in yeast mitochondria, purified and reconstituted into liposomes with an efficiency of ~80% (Supplementary Fig. 1). Uptake of radiolabelled ATP was measured in the presence or absence of different combinations of unlabelled substrates and effectors (Fig. 1a and b, and Supplementary Fig. 2). In the absence of internal ATP there was no discernable ATP uptake, regardless of the external calcium concentration, indicating that an internal counter substrate is required to complete the catalytic turnover at a detectable rate in this assay (Fig. 1a and b, and Supplementary Fig. 2). The highest rate of ATP uptake was observed for proteoliposomes loaded with ATP in the presence of external calcium, displaying a specific initial rate of ~100 nmol ATP min^−1^ mg^−1^. In the presence of EGTA, which removes calcium, the rate of ATP uptake dropped almost to background (Supplementary Fig. 2). There was no significant difference in ATP uptake between conditions in which no additional calcium was added or in which EGTA was added to the outside of the liposomes, suggesting that buffer exchange through the use of a PD10 column reduced calcium contaminations to levels that were unable to activate the carrier. Furthermore, there was no significant difference in the specific initial rate of ATP uptake catalysed by APC1 in the presence or absence of internal calcium. These results show that calcium-dependent activation could be observed for the isolated and reconstituted human APC1, demonstrating that it is fully active. Furthermore, only calcium added to the outside of the liposome had an effect on uptake activity suggesting that the regulatory domain is only accessible from the outside, and that the protein was reconstituted in an orientation that was predominantly with the regulatory domain on the outside of the proteoliposomes (~95%) (Fig. 1a and b, and Supplementary Fig. 2).

The external calcium concentration was titrated against liposome-reconstituted APC1 to study the effect on ATP transport and estimate the concentration required to half-maximally activate the protein (see Supplementary Fig. 3). However, key components of the assay, such as the lipids, can chelate calcium^24^, and therefore the free calcium concentration was estimated experimentally using a fluorescent calcium probe (Supplementary Fig. 3). After correction for bound calcium, the *EC*_50_ for free calcium was determined to be 179 ± 67 μM (Fig. 1e and Supplementary Fig. 3). The calcium sensitivity of various APC paralogues has been reported between 4 and 30 μM^6^,^11^,^21^, including a study using isolated mitochondria where human APC1 was thought to be the major isoform^11^. However, this is the first time that calcium dependency of ATP transport by the human APC1 has been assessed in a reconstituted system, showing that its activity will respond linearly to changes in cytosolic calcium concentrations.

Titration of EGTA on the outside of the liposomes, in the presence of 1 mM external calcium, showed that APC1 could be completely inactivated by the removal of calcium using EGTA concentrations above 1.5 mM (Supplementary Fig. 3). No further inhibition was observed with more EGTA additions, meaning that rates of ~5–10 nmol [^14^C]-ATP min^−1^ mg^−1^ protein represent the background signal, presumably due to slow leak or non-specific binding of the radiolabelled ATP to the liposomes.

In order to assess what type of regulatory effect calcium has on the kinetics of ATP transport catalysed by APC1, two different concentrations of calcium were added, one low calcium concentration below the calcium *EC*_50_ (0.25 mM total calcium) and one high calcium concentration (4 mM total calcium), and the effect on ATP transport measured (Fig. 1f and Supplementary Fig. 4). The *V*_max_ for ATP uptake was 194 (±22) nmol min^−1^ mg^−1^ and 1071 (±104) nmol min^−1^ mg^−1^ at 0.25 and 4 mM total calcium, respectively. Importantly, the apparent *K*_m_ was not significantly different (31 ± 9 μM and 29 ± 8 μM at 0.25 mM and 4 mM total calcium, respectively, Fig. 1f), demonstrating that the mechanism of calcium regulation is non-competitive with respect to ATP transport, as shown before for yeast APC^21^.

### The role of the amphipathic α-helix in calcium regulation

Thermostability assays have previously been used to probe conformational changes of mitochondrial carriers by assessing their thermostability in different states^25^,^26^,^27^. Here, they have been applied to determine the differences in thermostability between the calcium-bound and calcium-free state of detergent-solubilised APC1. Protein unfolding is monitored with the fluorescent dye 7-diethylamino-3-(4-maleimidophenyl)-4-methylcoumarin (CPM) that emits a fluorescent signal upon reaction with cysteine residues^28^. By using a controlled temperature ramp, thermal denaturation of the protein provides a melting curve, the inflection point of which is taken as the apparent melting temperature (*T*_m_) and used as a relative marker of protein stability^26^,^28^. APC1 has four cysteine residues: one in the regulatory domain and three in the carrier domain. We observed that residue C15 of the regulatory domain could be labeled with polyethylene glycol methyl ether maleimide 2000 Da (PEG-maleimide 2 K) in solution (Supplementary Fig. 5a), as labeling was abolished by a C15S mutation (Supplementary Fig. 5b). Therefore, C15 is already exposed in the native state before thermal denaturation, giving a raised baseline in the CPM assay (Fig. 1c). Only residues buried in the carrier domain were exposed and labeled during the thermal denaturation, thus reporting on the unfolding of the carrier domain only. In the absence of calcium, the *T*_m_ of APC1 was 9 °C higher than in the presence of calcium (Fig. 1c and d). This shift in stability to a higher *T*_*m*_ correlates with the calcium-free condition in which APC1 is inactive for substrate transport (Fig. 1c and d). Therefore, the thermostability assay is able to report on changes in stability of the carrier domain associated with calcium-induced activation or inhibition by the regulatory domain.

**Figure 2:**
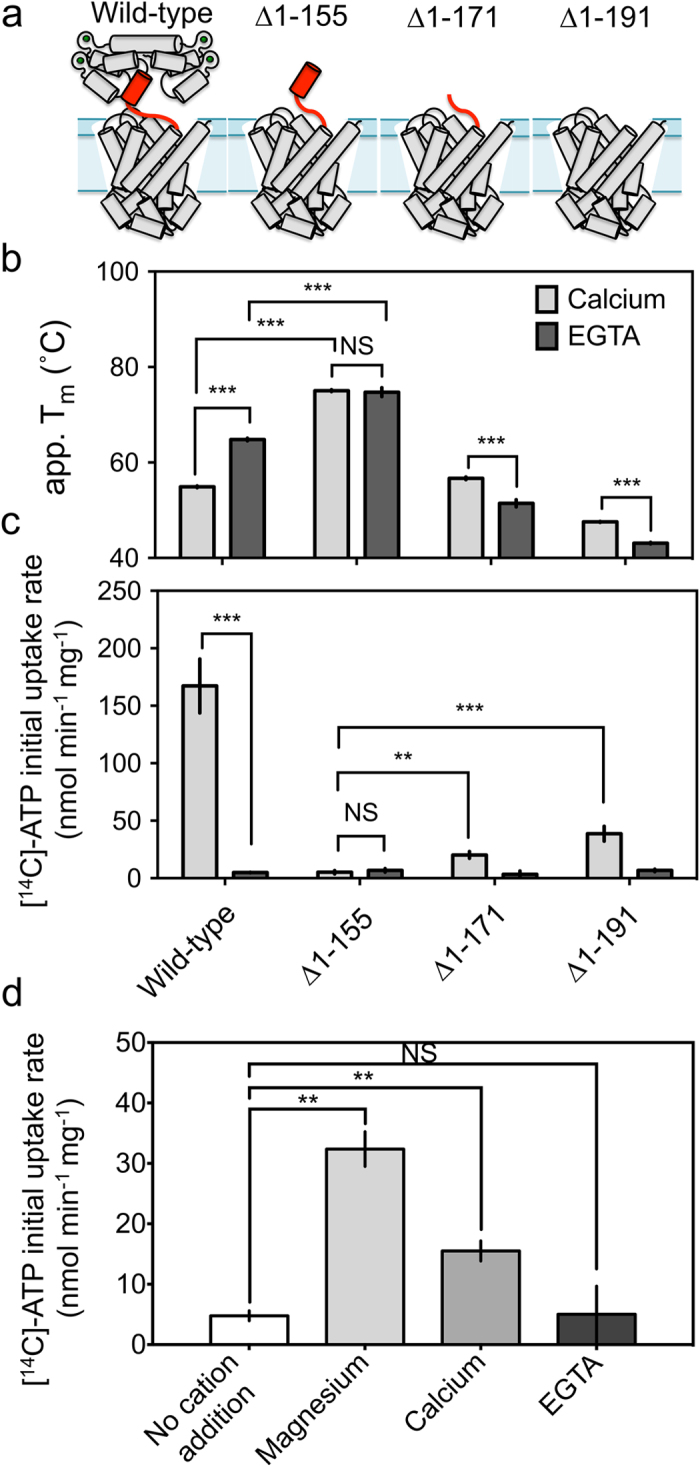
Thermostability and transport properties of wild-type and ∆1-155, ∆1-171 and ∆1-191 truncation mutants of APC1. (**a**) Cartoon representation of the tested truncation mutants. The amphipathic α-helix and linker-loop (residues 155-191) are indicated in red. (**b**) The apparent melting temperatures (app. *T_m_*) determined in the presence of 2.5 mM calcium chloride or EGTA, added prior to temperature ramping. (**c**) The specific initial uptake rate of 1.5 μM [^14^C]-ATP into proteoliposomes, containing wild-type, ∆1-155, ∆1-171 or ∆1-191 truncation mutants. The inside contained 2.5 mM ATP only (without calcium or EGTA), whereas on the outside there was 1.5 μM [^14^C]-ATP and 2.5 mM calcium chloride or 2.5 mM EGTA. (**d**) The N-terminal truncation (∆1-191_APC1) construct was reconstituted into liposomes and the amount of 1.5 μM [^14^C]-ATP taken up, measured. All liposomes were loaded with 2.5 mM ADP, 1 mM DTT and 20 mM tris-HCl pH 7.4. Either 5 mM magnesium chloride, 5 mM calcium chloride or 5 mM EGTA was added to the outside of the liposome, and hetero-exchange between ATP and ADP was monitored over a 15-minute time course. Specific initial rates were fitted and significance values were calculated using unpaired, two-tailed Student’s t-tests, where; NS p > 0.05; **p ≤ 0.01; ***p ≤ 0.001.

Previous analysis of the regulatory domain indicated that the amphipathic α-helix is a mobile element that binds to the regulatory domain in the presence of calcium, but might be released in the absence of calcium^12^. We postulate that the amphipathic α-helix interacts with the carrier domain in the calcium-free state in order to inhibit substrate transport. To investigate this possibility, a series of N-terminal truncations of the carrier were generated where the entire regulatory domain was removed, but where different parts of the linker region were still present. Truncation mutant ∆1-155 retains the amphipathic α-helix and linker loop, ∆1-171 retains just the linker loop, and ∆1-191 has no linker region at all (Fig. 2a). In all of the truncations, the calcium-dependent increases in *T*_*m*_ observed for wild-type APC1 were abolished (Fig. 2b). Instead, the truncation mutants ∆1-171 and ∆1-191 were slightly destabilised in the calcium-free condition, which may be a direct effect of EGTA on the stability of the carrier domain. Truncation mutant ∆1-155 APC1, which lacked only the regulatory domain, displayed a remarkable increase in *T*_*m*_ to 75.0 °C in the presence or absence of calcium (Fig. 2b). Proteoliposomes made with ∆1-155 truncation mutant did not display ATP uptake, whether calcium was present or not, indicating that this protein was constitutively inactive (Fig. 2c).

The ∆1-171 and ∆1-191 truncation mutants had stabilities of 56.7 and 47.5 °C in the presence of calcium, respectively, showing that ∆1-171 was equally and ∆1-191 was less stable than wild type in the presence of calcium. These results show that the amphipathic α-helix is the main contributor to the high thermostability of the ∆1-155 truncation mutant by interacting with the carrier domain and that the linker loop region provides interactions stabilising the carrier domain (see also below). Based on the thermostabilities of the ∆1-171 and ∆1-191 truncation mutants, we expected that both would be active, and indeed, in the presence of calcium, proteoliposomes made with ∆1-171 or ∆1-191 APC1 displayed ATP uptake activity, albeit to a lower level than the wild-type protein, which might be due to stability issues during reconstitution (Fig. 2c). Uptake of ATP by these truncation mutants was almost abolished in the presence of EGTA (Fig. 2c), which was surprising as the regulatory domain was missing. In order to understand this effect, ATP uptake was measured in ∆1-191 APC1 proteoliposomes in the presence of saturating conditions (5 mM) of magnesium chloride, calcium chloride or EGTA on the outside, in exchange for ADP loaded on the inside (we suspected that calcium was being transported). Without the addition of divalent cations to the outside of liposomes, the specific initial uptake rate of ATP was low (~5 nmol min^−1^ mg^−1^ protein; Fig. 2d). This low rate of ATP for ADP exchange was not due to the build-up of membrane potential (Supplementary Note 1 and Supplementary Fig. 6). The specific initial uptake rate of ATP was highest in the presence of externally added magnesium, increasing six-fold to ~30 nmol min^−1^ mg^−1^ protein (Fig. 2d). The addition of calcium also increased the specific initial uptake rate of ATP three-fold to ~15 nmol min^−1^ mg^−1^ protein (Fig. 2d), consistent with significant transport of ATP-Ca. Therefore, we concluded that the inhibitory effect of EGTA was due to the fact that free ATP is transported at much lower rates than ATP-Ca^23^ (see Supplementary Note 1 and Supplementary Fig. 6).

Our results refute the ‘capping mechanism’ involving the whole regulatory domain, as proposed previously^13^, which would predict full activity of ∆1-155 APC1. Instead these results demonstrate that the amphipathic α-helix is the key regulatory element, interacting with the carrier domain to inhibit substrate transport when released from the regulatory domain. We have named this a *locking pin* mechanism, as the amphipathic α-helix acts as a pin that is released by the regulatory domain in the absence of calcium, to bind and lock the carrier domain in an inhibited state.

### A structural model for the entire calcium-bound human ATP-Mg/Pi carrier

To understand the mechanism of calcium regulation in molecular detail, we constructed a model of the entire APC1 protein. A crystal structure of the calcium-bound regulatory domain^12^,^13^, and structural models of the calcium-free regulatory domain^12^ and carrier domain, based on the ADP/ATP carrier structures^16^,^19^, were available. However, a reference structure for the loop region of the protein between the regulatory and carrier domains (residues 171–191) was not, and so the position and orientation of the regulatory domain relative to the carrier domain was unknown. To aid model building we used the established bioinformatic method of evolutionary coupling analysis^29^. Evolutionary variation between APC orthologues was used to determine residue pairs that have co-evolved together because they might be spatially close to and/or interacting with one another. These residue pairs can be used as distance constraints for modeling. In total 470 potential interactions were provided by the EVfold server^29^, but we found that interactions with a pseudo-likelihood maximization score (PLM) below 0.2 could not be confirmed by the available structures or displayed spuriously large interaction distances (Supplementary Fig. 7 and Supplementary Table 1). Therefore, we restricted our analysis to EVcoupling pairs with PLM scores above 0.2, allowing us to build a network of interactions that were used as distance constraints in modeling (Supplementary Fig. 8 and Supplementary Table 2).

Many of the EVcoupling pairs could be confirmed in the available structural models, supporting the validity of the approach. Two evolutionary coupled pairs of residues (K96:N102 and N34:E43) were located in the regulatory domain, and their relative positions and interactions could be confirmed in the crystal structure (Supplementary Fig. 8c and d)^12^. Another four of the evolutionary coupled pairs of residues were located in the carrier domain (I435:L443, E438:R445, F238:L250 and F238:V424), and these interactions were consistent with the comparative homology model of the carrier domain (Supplementary Fig. 8e and f)^16^. The remaining 14 interacting pairs were located at the end of the amphipathic α-helix, in the linker-loop and at the N-termini of the first transmembrane helix of the carrier domain. The constraints supplied by the evolutionary coupling data allowed us to construct a model for the linker-loop region (Supplementary Fig. 8a and b). We noted that there were no co-evolved pairs between the regulatory domain and the carrier domain (Supplementary Fig. 8). Thus it appears that the regulatory domain interacts with the carrier domain via the linker-loop region, which would allow both domains to move independently of each other. It also shows that the linker-loop region is a key component of the overall structural stability of the transporter in agreement with the truncation analysis (Fig. 2). Given the lack of constraints, it was not possible to determine the lateral position of the regulatory domain relative to the carrier domain, but the constraints provided by the compact linker-loop domain did restrain the orientation of the regulatory domain relative to the carrier domain. A number of regulatory domain positions were modeled, and the highest scoring one was used, but all are in agreement with the *locking pin* mechanism (Supplementary Fig. 8a and b).

### State-dependent thermostability shifts in ATP-Mg/Pi carrier mutants

**Figure 3:**
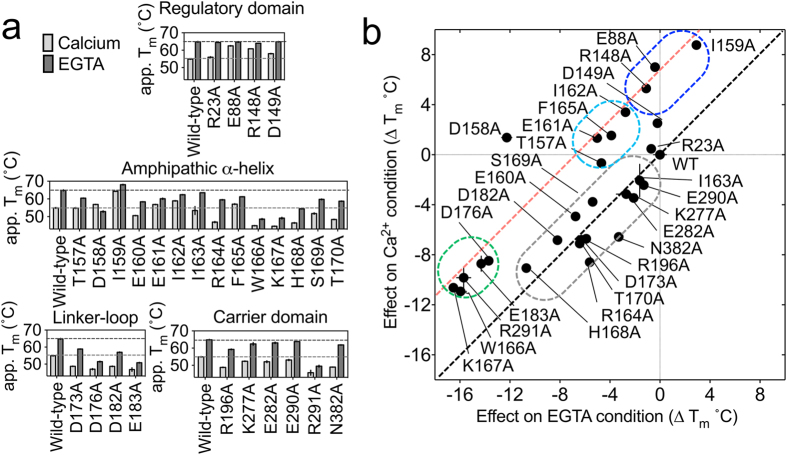
Effect of mutations on the thermostability of APC1. (**a**) Effect of mutations on the apparent thermostability of APC1. The apparent melting temperatures of 28 alanine replacement mutants were determined by using the CPM thermostability assay. Either 2.5 mM calcium chloride (light gray bars) or EGTA (dark gray bars) were added to the sample 10 minutes before temperature ramping. (**b**) The difference in *T*_*m*_ of each mutant from the *T*_*m*_ of wild-type APC1 was calculated. For each mutant, the ∆*T*_*m*_ in the calcium condition was plotted against the ∆*T*_*m*_ in the EGTA condition. Mutations that have a similar effect on stability to one another in both the presence and absence of calcium cluster together on the graph, and have been grouped accordingly with a colored line. Mutations on the diagonal line (black dashed line) have a proportional effect on stability in the presence and absence of calcium. Mutations on the parallel line (red dashed line), shifted by 9 °C relative to the wild-type, have a disproportional effect on stability in one condition over the other, indicating an effect on regulation.

Next we set out to investigate the role of specific residues to probe the molecular detail of the *locking pin* mechanism. We were particularly interested in residues of the amphipathic α-helix, the regulatory domain and the cytoplasmic side of the carrier domain. As the mechanism of calcium regulation is likely to be universally conserved across orthologues, we selected conserved residues for mutagenesis by inspecting sequence alignments of APC orthologues (Supplementary Fig. 9). Charged residues of the carrier and regulatory domain that are conserved in more than 50% of the orthologues were selected, but those playing a role in calcium binding^12^,^13^ or substrate transport^17^,^18^ were excluded, leaving a set of 14 residues (Supplementary Fig. 9). All 14 residues of the amphipathic α-helix were also selected for mutation. Thus, in total 28 single alanine replacements were made and all of the mutant full-length carriers were expressed in yeast mitochondria, solubilised and purified. The thermostability of the mutant proteins was analysed in the presence or absence of calcium (Fig. 3a). In all cases, typical sigmoidal melting profiles were obtained, confirming the presence of folded protein and allowing *T*_*m*_ values to be determined (Fig. 3a).

To interpret these results, the *T*_*m*_ of wild-type was subtracted from the *T*_*m*_ of each mutant, providing a relative difference in melting temperature (Δ*T*_*m*_) in both the calcium-bound (active) and calcium-free (inactive) states. The differences in the presence of calcium were plotted against those in the presence of EGTA (Fig. 3b). Mutations with a similar effect on thermostability in both the calcium-bound and calcium-free states point to residues that are equally important for protein stability in both states, indicating a similar structural role in both conditions (black diagonal line - Fig. 3b). Mutations that affected the thermostability in one state more than the other point to residues that are likely to be involved in calcium regulation. In Fig. 3b, these mutants are shifted to the red parallel line, which represents the position of thermostabilities with a complete absence of calcium-dependent changes, i.e. a loss of the ~9 °C difference in thermostability between the calcium-bound and calcium-free state, as observed for the wild-type protein (Fig. 1a and d). Mutations with similar effects clustered into four distinct groups (colored dashed rings in Fig. 3b).

Representative members of the four clusters were assessed for their ability to transport substrates in both the presence and absence of calcium. Mutations at D158 (no cluster), E161 (cyan cluster) and K277 (grey cluster) responded to calcium and EGTA in a similar way to wild-type, however, mutations at E88 and I159 (blue cluster) resulted in a significantly lower uptake activity in the presence of calcium in comparison to wild-type (Supplementary Fig. 10). Uptake assays were attempted on the D176A, E183A and R291A mutants (green cluster), but their transport properties could not be determined, because their overall thermostability was much lower than that of the wildtype and blue cluster mutants (Fig. 3a), which lead to stability issues during reconstitution.

### Role of individual residues in the mechanism of calcium regulation

The blue and green clusters of mutants must be important for the regulatory mechanism, but they differ fundamentally from each other in the way the mutations affect the thermostability. The blue cluster is predominantly affected in the calcium condition, being ~9 °C more stable (vertical shift), whereas the green cluster is predominantly affected in the EGTA condition, being ~9 °C less stable (horizontal shift) (Fig. 3b). Accordingly, the blue cluster mutants should be shifted towards a constitutively inactive state, while the green cluster mutants should be shifted toward a constitutively active state. The transport activity of the blue cluster mutants I159A and E88A in the presence of calcium correlated inversely with their thermostability shift; the more the residue stabilised the calcium-bound state, the bigger the inactivation of transport upon mutation (i.e. mutations further up the red dashed line are those most impaired in regulatory activity; Supplementary Fig. 10), consistent with an increased likelihood of amphipathic α-helix release, which leads to inhibition of the carrier.

**Figure 4:**
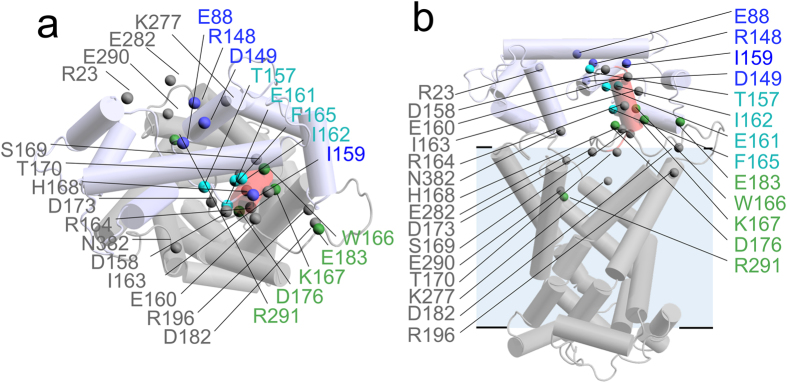
Positions of the 28 alanine replacements mapped to the APC1 model. (**a**) View from the membrane and (**b**) view from the cytoplasmic side of the APC1 model. The mutations are shown as spheres and they are colored according to clusters identified in Fig. 3b. The regulatory domain (blue) is based on the APC1 regulatory domain crystal structure (PDB: 4ZCV)^12^. The carrier domain (grey) is a comparative homology model based on the crystal structure of bovine AAC (PDB: 1OKC)^16^,^49^. The linker-loop structure is based on evolutionary coupling data (Supplementary Fig. 8 and Supplementary Table 2).

**Figure 5:**
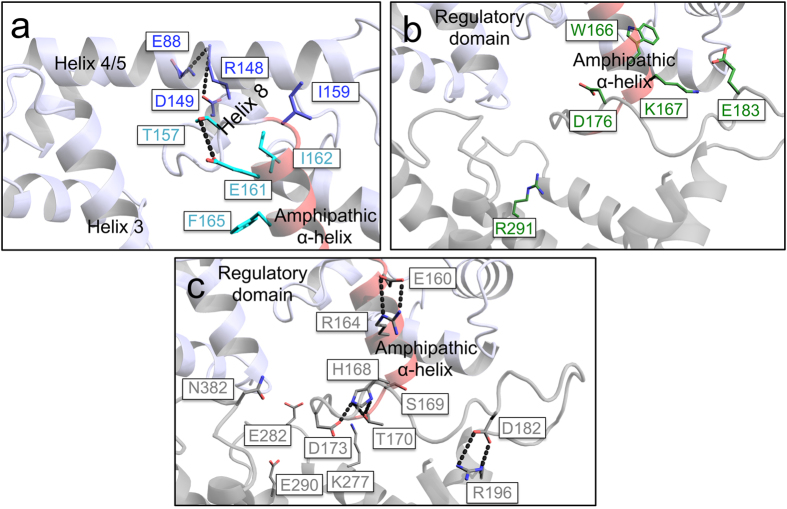
Interactions between residues highlighted by the thermostability shift assays. Detailed views of residues of (**a**) the blue and cyan clusters, (**b**) the green cluster and (**c**) the grey cluster of mutants (Fig. 3b). Possible hydrogen bond interactions are displayed as dotted black lines. The entire APC1 model has been constructed as explained in Supplementary Fig. 8.

In order to interpret the role of each cluster of mutants, their structural positions were mapped on to a model of the calcium-bound APC1 (Fig. 4a and b). The mutations E88A, R148A and D149A of the blue cluster have similar effects on stability (Fig. 3b), and they are close in the structure of the regulatory domain^12^, forming a salt-bridge network, which we have named the ‘ERD-clasp’ (Fig. 5a). Similarly, mutations T157A, E161A, I162A and F165A of the cyan cluster are also close to each other in the regulatory domain^12^, but are at the N-terminus of the amphipathic α-helix (Fig. 5a). In descending order, mutations at residues I159, E88 and R148 display the largest thermostability shifts from wild-type (Fig. 3b) and, when tested, they display an impairment in ATP uptake that correlates with the severity of the thermostability changes (Supplementary Fig. 10). Therefore, all of these residues play an important role in stabilising the active state of the protein, because they are involved in binding of the amphipathic α-helix to the regulatory domain in agreement with the available structural information.

The results above show that residues that are close to one another, performing a similar structural/functional role, cluster together in the thermostability shift assays. The residues of the green cluster show behavior we would expect for those involved in the calcium-free inactive state of the protein when the amphipathic α-helix interacts with the carrier domain. Residues W166, K167, D176, and E183 (green cluster in Fig. 3b) are all found at the C-terminal end of the amphipathic α-helix and linker-loop (Fig. 5b). Residue R291, which is located in the carrier domain, gave a similar effect on thermostability in the calcium-free state as residues D176 or E183 of the amphipathic α-helix, and it is possible that it interacts with them in the inhibited state.

Mutations with a similar effect on thermostability in both the calcium-bound and calcium-free states are residues that are equally important for protein stability in both conditions, indicating that they are not involved in regulation. Mutations of residues E160, R164, H168, S169, T170, D173, D182, R196 and N382 (grey cluster in Fig. 3b) belong to this category and are located at the end of the amphipathic α-helix, throughout the linker-loop and at the cytoplasmic side of the carrier domain (Fig. 4a and b). These residues were independently positioned close to one another in our model based on evolutionary coupling (Fig. 5c), showing again consistency between the evolutionary coupling and thermostability data. It is likely that the residues in the grey cluster are involved in stabilising interactions important for the structural integrity of the protein, forming a folded domain between the regulatory domain and carrier domain.

Residue D158 appears to be an outlier in the thermostability shifts (Fig. 3). This residue is not conserved, and the level of inhibition was not significantly different from that of the wild-type in transport experiments (Supplementary Fig. 10). Furthermore, this residue is not involved in any interactions with the regulatory domain in the calcium-bound regulatory domain crystal structure of APC1^12^. Therefore, D158 may be involved in the regulatory mechanism, but we cannot assign a role for it.

### Proposed conformational changes between the calcium-bound and calcium-free states

**Figure 6:**
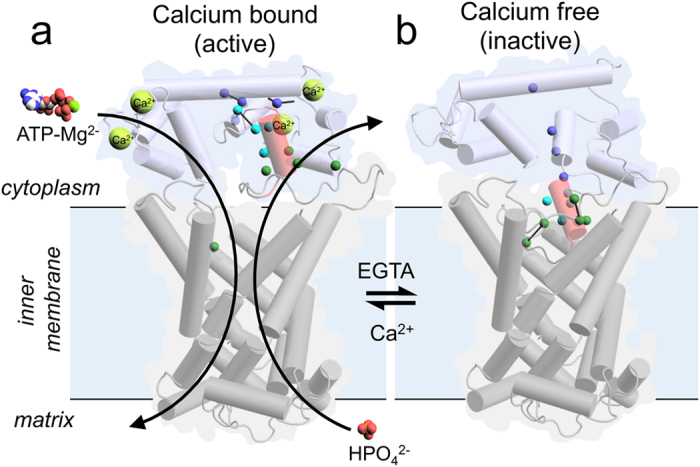
Role of the amphipathic α-helix in the proposed *locking pin* mechanism of calcium regulation. View from the membrane of the (**a**) calcium-bound or (**b**) calcium-free states of the APC1 model. The calcium-bound regulatory domain in blue is based on PDB: 4ZCV. Residues of the blue and green clusters are displayed as colored spheres, representing the Cα atoms. Known (**a**) and predicted (**b**) interactions between residues in the different states have been indicated with dashed black lines. The calcium-free regulatory domain is based on a model in which all EF-hands of the regulatory domain are closed^12^. The carrier domain in grey is a comparative model based on the bovine AAC crystal structure (PDB: 1OKC)^16^. The amphipathic α-helix is shown in red. The α-helices are represented as cylinders. The substrates ATP-Mg and phosphate, and activating calcium ions are in sphere representations.

Based on all of our results, we have constructed a model for the calcium-free state of APC1 and propose a conformational change between the two states (Fig. 6 and Supplementary Movie 1). In this calcium-free model, the EF-hands of the regulatory domain are closed, excluding the amphipathic α-helix from its hydrophobic binding pocket in the regulatory domain^12^. Instead, the amphipathic α-helix interacts with residues of the cavity in the carrier domain, inhibiting its function (Fig. 6). In the calcium-bound model, the residues of the blue cluster are close to one another and engaged in bonding interactions, facilitating the locking of the amphipathic α-helix into its hydrophobic binding pocket in EF-hands 3 and 4, and allowing the carrier domain to be active. The amphipathic α-helix is released in the calcium-free state and these residues are no longer close to one another (Fig. 6). In contrast, residues of the green cluster are far apart in the calcium-bound model, but move closer together when the amphipathic α-helix binds to the carrier domain (Fig. 6).

## Discussion

Normally EF-hand mechanisms involve the binding of an amphipathic α-helix in a target polypeptide by a calcium-bound EF-hand, propagating downstream signaling events^30^. Here, the *locking pin* mechanism shares features of this mechanism, but differs also, as the regulatory amphipathic α-helix and EF-hands are part of the same polypeptide chain. It represents an interesting example of evolution in which different domains have been combined in order to regulate substrate transport. There are other examples of EF-hand-containing proteins that have this type of ‘self-sequestered’ interaction^31^,^32^,^33^,^34^, but only the mitochondrial aspartate/glutamate carrier (AGC) has a similar combination of domains^35^. AGC also displays a calcium-dependent stimulation of activity in its carrier domain^36^,^37^ when calcium binds to its EF-hand containing N-terminal regulatory domain^35^. Even so, APC and AGC do not share significant sequence similarity in their regulatory domains; APC has canonical EF-hand motifs like calmodulin, whereas AGC contains S100-like calcium-binding motifs^12^,^35^. In addition, the AGC regulatory domain contains a dimerisation interface for the formation of homo-dimers^35^, whereas APC is monomeric^12^. Furthermore, in AGC the carrier domain is inserted between the EF-hands and the target α-helix, whereas in APC the target α-helix is inserted between the EF-hands and the carrier domain. The proposed regulatory mechanism of AGC involves rotation of a mobile unit of EF-hands 1–2 against the static unit of EF-hands 4–8 of the regulatory domain to close access to the carrier domain^35^. Here, we show that the regulatory mechanism of APC is fundamentally different in which EF-hands have evolved in a unique way to regulate substrate transport by a *locking pin* mechanism.

## Methods

### Sequence alignment

The Basic Local Alignment Search Tool^38^ was used to search for orthologues of either the *Saccharomyces cerevisiae* or *Homo sapiens* APC sequences. Over 300 unique APC sequences of putative APC orthologues from a diverse range of mammalian, invertebrate, plant and fungal species were collated. Initial alignment between the sequences was carried out using ClustalW^39^. Representative sequences from each phylogenetic clade were selected for manual alignment and refinement. In this way the list of sequences was manually curated to produce a core set of ~30 aligned sequences that accurately represent the conservation and diversity of APC sequences across all phyla. The sequence alignment combined with secondary structure prediction tools (PSIPRED)^40^ were used in order to predict the secondary structure elements.

### Cloning and mutagenesis

A construct of APC1 (Uniprot: Q6NUK1) was designed to include an eight-histidine tag followed by a Factor Xa cleavage site (IEGR) at the N-terminus, creating a cleavable purification-tag. The gene was codon-optimised for expression in *S. cerevisiae* (GenScript, Piscataway, USA) and cloned into a pYES3/CT expression plasmid (Invitrogen, Paisley, UK), with the inducible galactose promoter replaced by the constitutive promoter for the yeast mitochondrial phosphate carrier by a method described previously^41^.

The method of overlap extension PCR was used in order to introduce 28 specific point mutations into the APC1 gene sequence^42^. Three truncations of the HsAPC-1 construct were also prepared utilising three different forward primers for the ∆1-155 truncation (GAATTCAACATGTCTCACCACCACCACCACCACCACCACTCCGACGCCGCCATAGAAGGTAGAACCTCCGAAGATGTTACTGATATCGAAGAAATCATCAG), the ∆1-171 truncation (GAATTCAACATGTCTCACCACCACCACCACCACCACCACTCCGACGCCGCCATAGAAGGTAGAACCTCCGAAGATGGTATTGATATAGGTGACAGTTTAAC) and the ∆1-191 truncation (GAATTCAACATGTCACATCACCATCATCATCATCATCACTCAGATGCTGCTATTGAAGGTCGTACATCAGAAGATTCTGGTCAATGGTGGAGACAATTGTTAGC), and all truncations used the same reverse primer (CCTAGGTCTAGACTCGAGTCA). The wild-type APC1, mutant and truncated constructs were ligated into the expression plasmid and then transformed into *S. cerevisiae* haploid strain W303-1b^43^, using established methods^44^.

### Expression in yeast

*S. cerevisiae* was cultured, and the truncated and mutated APC1 were expressed following established methods^19^, with the following modifications: pre-cultures of yeast were set up in synthetic-complete tryptophan-dropout medium (Formedium, Hunstanton, UK) supplemented with 2% glucose, and main cultures were carried out in 10 × 2.5 L full-baffle TunAir® shake flasks (Sigma-Aldrich, Gillingham, UK) with 1 L of YPD medium in each. Mitochondria were prepared using established methods^45^, flash frozen in liquid nitrogen, and stored under liquid nitrogen until use.

### Solubilisation and purification

For the purification of wild-type, mutant and truncated APC1, the protein was solubilised in 1.5% lauryl maltose neopentyl glycol (1 hour, 4 °C), and separated from insoluble protein by centrifugation (182,000 × g, 1 hour, 4 °C). Solubilised protein was then passed through nickel sepharose affinity resin (GE Healthcare, Little Chalfont, UK) at 1 mL min^−1^ (ÄKTA prime, 4 °C) to selectively bind APC1. The affinity resin was washed with 50 column volumes of buffer A (20 mM tris-HCl, pH 7.4, 150 mM sodium chloride, 20 mM imidazole, 0.1% (w/v) lauryl maltose neopentyl glycol, 0.1 mg mL^−1^ tetraoleoyl cardiolipin) and 30 column volumes of buffer B (20 mM tris-HCl, pH 7.4, 50 mM sodium chloride, 0.1% (w/v) lauryl maltose neopentyl glycol, 0.1 mg mL^−1^ tetraoleoyl cardiolipin). 120 units of factor Xa protease (New England Biolabs, Ipswich, USA) was used to specifically cleave APC1 protein from the affinity resin (18 hours, 4 °C). After factor Xa cleavage, APC1 was spun (1,000 × g, 3 minutes, 4 °C) through an empty Proteus midi-spin column (Generon, Maidenhead, UK) to remove affinity resin.

### Reconstitution

The lipids L-α-phosphatidylcholine (Avanti Polar Lipids, Alabaster, USA) and tetraoleoyl cardiolipin (Avanti Polar Lipids, Alabaster, USA) were mixed in a 20:1 (w/w) ratio and dried under a stream of nitrogen. Lipids were washed once with methanol before being dried once again in a stream of nitrogen. Lipids were re-hydrated in 20 mM tris-HCl pH 7.4, 1 mM DTT to a final lipid concentration of 12 mg mL^−1^. Cold substrates or effectors to be internalised into the interior of liposomes were added at this point as concentrated stocks. 2% (v/v) of the detergent pentaethylene glycol monodecyl ether was added and the lipids were solubilised by vortexing. Protein was added to the sample in a 100:1 (w/w) ratio, lipid to protein, diluting the protein 10-fold to a concentration of 0.1 mg mL^−1^. The samples were then incubated with inversion at 4 °C for one hour. The pentaethylene glycol monodecyl was removed, and liposomes formed by SM-2 bio-bead (Bio-Rad, Hemel Hempstead, UK) additions. Eight additions of bio-beads were made to the sample, the first four of 60 mg, and the final four of 120 mg. Between additions, the samples were incubated with inversion at 4 °C for 20 min each. Following the final bio-bead addition the samples were incubated overnight at 4 °C with inversion. Bio-beads were removed by passage of the sample through empty micro-bio spin columns (Bio-Rad, Hemel Hempstead, UK). The external buffer was replaced with 20 mM tris-HCl pH 7.4 using a PD10 desalting column (GE Healthcare, Little Chalfont, UK), taking care to prevent carry over of cold substrates or effectors. The sample was diluted with assay buffer for a final lipid concentration of 1 mg mL^−1^.

### Uptake assays

Uptake assays were carried out using a Hamilton MicroLab Star robot (Hamilton Robotics Ltd., Birmingham, UK). Uptake of radiolabeled ATP was initiated at room temperature (20 °C) by the addition of 100 μL 20 mM tris-HCl, pH 7.4 buffer with 1.5 μM [^14^C]-ATP (2.035 GBq mmol^−1^; Moravek, Brea, USA) to 100 μL of liposomes plus effectors (calcium, magnesium or EGTA) in a MultiScreenHTS-HA 96-well filter plate (pore size, 0.45 μm; Millipore, Billerica, USA). The transport was stopped at 0, 10, 20, 30, 45 s, 1, 2.5, 5, 7.5, 10 and 15 min by the addition of 200 μL ice-cold 20 mM tris-HCl buffer and filtering using a vacuum manifold, followed by an additional wash step with 200 μL ice-cold 20 mM tris-HCl, pH 7.4 buffer. Levels of radioactivity in the liposomes were measured by the addition of 200 μL MicroScint-20 (Perkin Elmer, Waltham, USA) and by quantifying the amount of radioactivity with the TopCount scintillation counter (Perkin Elmer, Waltham, USA). Specific initial uptake rates were calculated using the amount of protein loaded into the liposome, which was quantified by SDS-PAGE. In the assays the final concentration of lipid and protein was 0.5 mg mL^−1^ and about 25 μg mL^−1^ (but specifically quantified for each sample), respectively.

Curves for each uptake were fitted following a one-phase association relationship in Prism (GraphPad). Initial rates were determined from the fit of the entire curve over the 15 minutes. There was a slight delay between the addition of radioactive substrate for the zero time point and the washing and vacuuming steps. For this reason the value was not constrained to zero during curve fitting, allowing a better fit of the data, and the delay was determined to be approximately 15 s, consistent throughout the entire series of experiments.

In order to estimate the free calcium concentration in liposome samples where different total calcium concentrations had been added, fluorescence readings (ex/em; 494/536 nm) were taken using a Spectramax M2 (Molecular Devices, Sunnyvale, USA) plate reader after the addition of 1 μM of the low affinity calcium probe fluo-5N (Thermo Fisher Scientific, Waltham, USA). Fluorescent signal was compared to a standard curve at 0.2, 1, 5, 10, 30, 200, 1000 μM calcium chloride in 20 mM tris-HCl pH 7.4, and the free-calcium concentration interpolated from the fit of the standard curve. Curves were fitted describing hyperbolic saturation kinetics for each set of initial uptake rates that had been corrected for free calcium independently providing three independent estimates of the *V*_max_ and *EC*_50_.

Each *N* number represents an independent liposome preparation and error bars represent the standard deviation between them. Statistical tests were calculated using unpaired, two-tailed Student’s *t*-tests, where; NS *P* > 0.05; *P ≤ 0.05; **P ≤ 0.01; ***P ≤ 0.001.

### PEG-maleimide assay

0.6 μg of either wild-type or C15S APC1 was diluted into 500 μL of assay buffer (20 mM tris pH 7.4, 0.1% lauryl maltose neopentyl glycol, 0.1 mg mL^−1^ tetraoleoyl cardiolipin). The sample was split into two aliquots, and to one, 0.2% (w/v) SDS was added. For a non-labelled sample, 25 μL from each was removed before reaction with polyethylene glycol methyl ether maleimide 2000 kDa (PEG-maleimide 2 K; Sigma-Aldrich, Gillingham, UK). A further 25 μL sample to act as a control for DTT quenching; to which 20 mM DTT was added before adding 0.5 mM PEG-maleimide 2 K. For the main reaction, 0.5 mM PEG-maleimide 2 K was added to the sample, 25 μL aliquots taken at 1, 5, 10, 20 and 30 min and quenched immediately with the addition of 20 mM DTT. Each of the samples were then mixed with loading buffer and analysed by SDS-PAGE.

### Thermostability assays

A fluorescence-based procedure for the assessment of detergent-solubilised and purified membrane protein stability was used, following established methods^26^,^28^. Briefly, 5 mg mL^−1^ stocks of 7-diethylamino-3-(4-maleimidophenyl)-4-methylcoumarin (CPM) dissolved in DMSO were diluted 50-fold into the assay buffer (20 mM tris-HCl pH 7.4, 20 mM NaCl, 0.1 mg mL^−1^ tetraoleoyl cardiolipin, 0.1% (w/v) lauryl maltose neopentyl glycol) and incubated for 10 min at room temperature. For each test, purified protein was diluted 50-fold in the same assay buffer (approximately 50 μg mL^−1^), and incubated on ice for 10 min in 200 μL thin-walled PCR tubes. 5 μL of the CPM dilution was added to the sample to make a final volume of 50 μL and incubated on ice for a further 10 min. The samples were subjected to a high-resolution melt (HRM) procedure on a Rotor-Gene Q 2plex HRM qPCR cycler with a 36-sample rotor (Qiagen, Venlo, the Netherlands).

In the assay, buried cysteine residues become solvent-exposed as protein unfolds, and react with CPM to form fluorescent adducts (ex/em optima of 387/463 nm). Protein unfolding profiles were analysed using the Rotor-gene Q software and the peak in the derivative of the fluorescence signal as a function of temperature, the ‘melt’ temperature (*T*_*m*_), provided a relative measure of protein stability.

### Protein analysis

Protein concentrations were determined using the bicinchoninic acid assay^46^ against a bovine serum albumin standard curve (Pierce, Loughborough, UK). The proteoliposomes used for uptake assays were loaded onto an SDS-PAGE gel and the protein concentration estimated by comparison to a standard curve of APC1 of known concentration. SDS-PAGE gels were composed of 12% acrylamide and run using a tris-glycine buffer system. Gels were loaded with 5:1 mix of sample to loading buffer (10% SDS (w/v), 1% bromophenol blue (w/v), 50% glycerol (v/v), 500 mM DTT, 250 mM tris-hydrochloride pH 6.8). Gels were stained with Imperial coomassie stain (Bio-Rad, Hemel Hempstead, UK) for one hour, and de-stained overnight in water.

### APC1 model building

Interacting pairs of co-evolved residues were identified using EVfold web server^29^. In total 1844 APC sequences were identified by the program, aligned and compared using EVfold server default options. The program identified 470 possible paired residues, and ranked them using a pseudo-likelihood maximisation (PLM) scoring method^29^. Of these pairs, only those with a PLM score greater than 0.2 were considered. This was the range in which pairs with known structural positions could be used to validate correctly identified evolutionary couples. Of the 20 residue pairs that were considered, 14 were in the linker-loop region of unknown structure and five of those were between residues that could form hydrogen bond interactions (Supplementary Table 1).

Five possible hydrogen bond interactions between evolutionary coupled residues were used as distance constraints in model building (Supplementary Table 1). The I-TASSER web server^47^ was used to build an initial structure of residues 170-193, using specified distances of 3 Å for hydrogen bond interactions. The model that best agreed with the distance constraints was used, which predicted a compact structure rather than a fully extended loop domain. The distance between the C-terminus of the regulatory domain and the N-terminus of the carrier domain could be constrained to the length of a compact linker-loop, providing a relative orientation of the regulatory domain to the carrier domain. However, the lateral position of the regulatory domain relative to the carrier could not be fixed. Many possible poses of the regulatory domain relative to the carrier domain were modeled in order to construct a model for the entire APC1 protein^48^ and each was energy-minimised using the MODELLER web server^49^. The highest scoring model was used as a reference structure and run with the I-TASSER web server^47^, specifying distance restraints once again. Some manual improvements were made to output models to improve distances between interacting residues, for example selecting correct side-chain rotamers^48^. The final distances between interacting residues are displayed in (Supplementary Table 1). It should be noted that all models positioned the amphipathic α-helix close to the cavity of the carrier domain consistent with the proposal that it interacts with the carrier domain in a *locking-pin* mechanism, but the highest scoring model was chosen in order to represent the full-length protein.

The final model has been submitted to the protein model database under accession code: PM0080481.

## Additional Information

**How to cite this article**: Harborne, S. P. D. *et al*. Calcium regulation of the human mitochondrial ATP-Mg/Pi carrier SLC25A24 uses a locking pin mechanism. *Sci. Rep.* **7**, 45383; doi: 10.1038/srep45383 (2017).

**Publisher's note:** Springer Nature remains neutral with regard to jurisdictional claims in published maps and institutional affiliations.

## Supplementary Material

Supplementary Movie 1

Supplementary Information
