# Supplementary material for: Calcium regulation of the human mitochondrial ATP-Mg/Pi carrier SLC25A24 uses a locking pin mechanism
Source: Sci Rep. 2017 Mar 28;7:45383. doi: 10.1038/srep45383 (PMC5369052; doi:10.1038/srep45383)
Supplement: Supplementary Information [file srep45383-s2.pdf]

Calcium regulation of the human mitochondrial ATP-Mg/Pi carrier SLC25A24 uses a locking pin mechanism

Steven P.D. Harborne, Martin S. King, Paul G. Crichton and Edmund R.S. Kunji

Supplementary Note 1:

Substrate level effects of magnesium and calcium on ATP transport

Divalent cations can act as transport substrates as well as regulators[1,2], and so it was important to separate the two processes. An APC1 construct in which the entire regulatory domain was removed (Δ1-191_APC1), was expressed, solubilised, purified and reconstituted into liposomes. Removal of the entire regulatory domain (Δ1-191_APC1) allowed the transport specificity of the carrier domain for divalent cations to be assessed, without the confounding influence of regulation. Furthermore, as it is believed that $[ATP\text{-}Mg]^{2-}$ is the preferred substrate of APC to allow electroneutral hetero-exchange with HPO_4^{2-} or $[H\text{-}ADP]^{2-}$ [3], we wanted to set up a net exchange of charge using an ATP/ADP hetero-exchange to characterise the effects of calcium, magnesium and membrane potential on substrate transport carried out by the carrier.

The Δ1-191_APC1 was expressed, purified and reconstituted into liposomes and ATP uptake was measured in saturating conditions (5 mM) of magnesium chloride, calcium chloride or EGTA on the outside, in exchange for ADP loaded on the inside (Supplementary Fig. 6). Without the addition of divalent cations to the outside of liposomes, the specific initial uptake rate of ATP was low (~5 nmol min^{-1} mg^{-1} protein). This low rate of ATP for ADP exchange was not due to the build up of membrane potential by the exchange of a minus 3 species for minus 2 species, as there was no significant difference in rate in the presence of valinomycin and potassium salts, which will dissipate the membrane potential (Supplementary Fig. 6). The specific initial uptake rate of ATP was highest in the presence of externally added magnesium, increasing six-fold to ~30 nmol min^{-1} mg^{-1} protein (Supplementary Fig. 6). The addition of calcium also increased the specific initial uptake rate of ATP three-fold to ~15 nmol min^{-1} mg^{-1} protein (Supplementary Fig. 6). These results confirm that uptake of ATP by APC has a requirement for ATP chelated to magnesium, but also suggest that ATP chelated to calcium is also preferably transported over free ATP.

Supplementary References

1. Fiermonte, G. *et al.* Identification of the mitochondrial ATP-Mg/Pi transporter. Bacterial expression, reconstitution, functional characterization, and tissue distribution. *J. Biol. Chem.* **279,** 30722–30730 (2004).
2. Lorenz, A. *et al.* In vitro analyses of mitochondrial ATP/phosphate carriers from Arabidopsis thaliana revealed unexpected Ca^{2+}-effects. *BMC Plant Biol.* **15,** 238 (2015).
3. Satrústegui, J., Pardo, B. & del Arco, A. Mitochondrial transporters as novel targets for intracellular calcium signaling. *Physiol. Rev.* **87,** 29–67 (2007).
4. Waterhouse, A. M., Procter, J. B., Martin, D. M. A., Clamp, M. & Barton, G. J. Jalview Version 2--a multiple sequence alignment editor and analysis workbench. *Bioinformatics* **25,** 1189–1191 (2009).
5. Hopf, T. A. *et al.* Sequence co-evolution gives 3D contacts and structures of protein complexes. *Elife* **3,** 1–45 (2014).

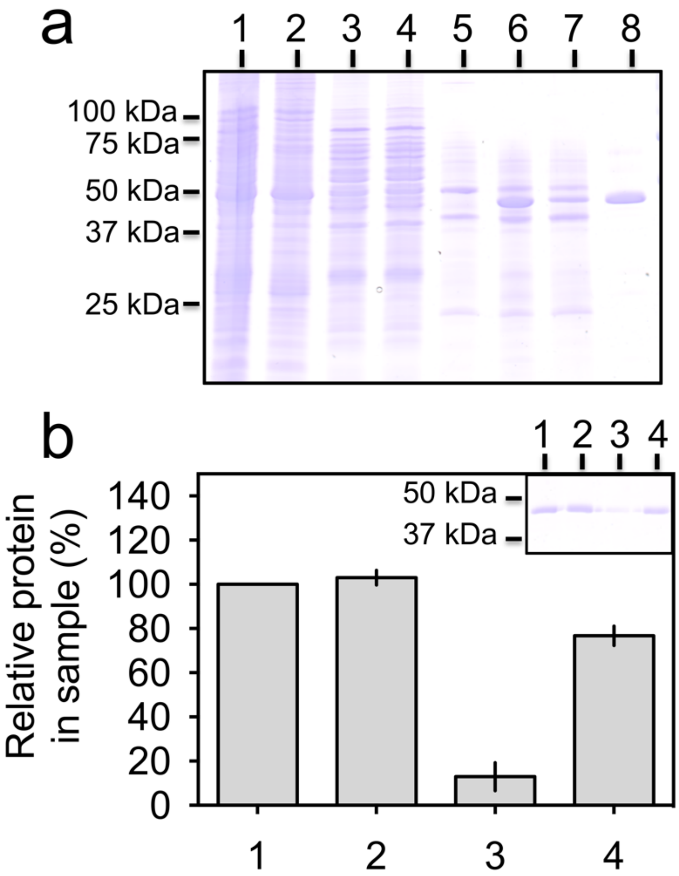

Supplementary Fig. 1 Purification and reconstitution of APC1

a) 12 % acrylamide gel of APC1 purification: 1, Isolated yeast mitochondria; 2, pellet after ultracentrifugation (unsolubilised protein); 3, soluble protein in detergent; 4, proteins that do not bind to Ni-sepharose; 5, protein bound to Ni-sepharose; 6, protein and Ni-sepharose resin after factor Xa treatment; 7, protein that remains bound to resin after separation through a centrifugation filter; 8, protein that is eluted after factor Xa treatment (purified carrier). b) Quantification of APC1 by SDS-PAGE (inset): 1, total protein at the start of reconstitution; 2, protein after the removal of detergent and desalting; 3, protein not associated with liposomes after high speed spin; 4, protein associated with liposomes after high speed spin, indicating that approximately 80 % of the protein was incorporated. Error bars represent the standard deviation from three independent gel lanes.

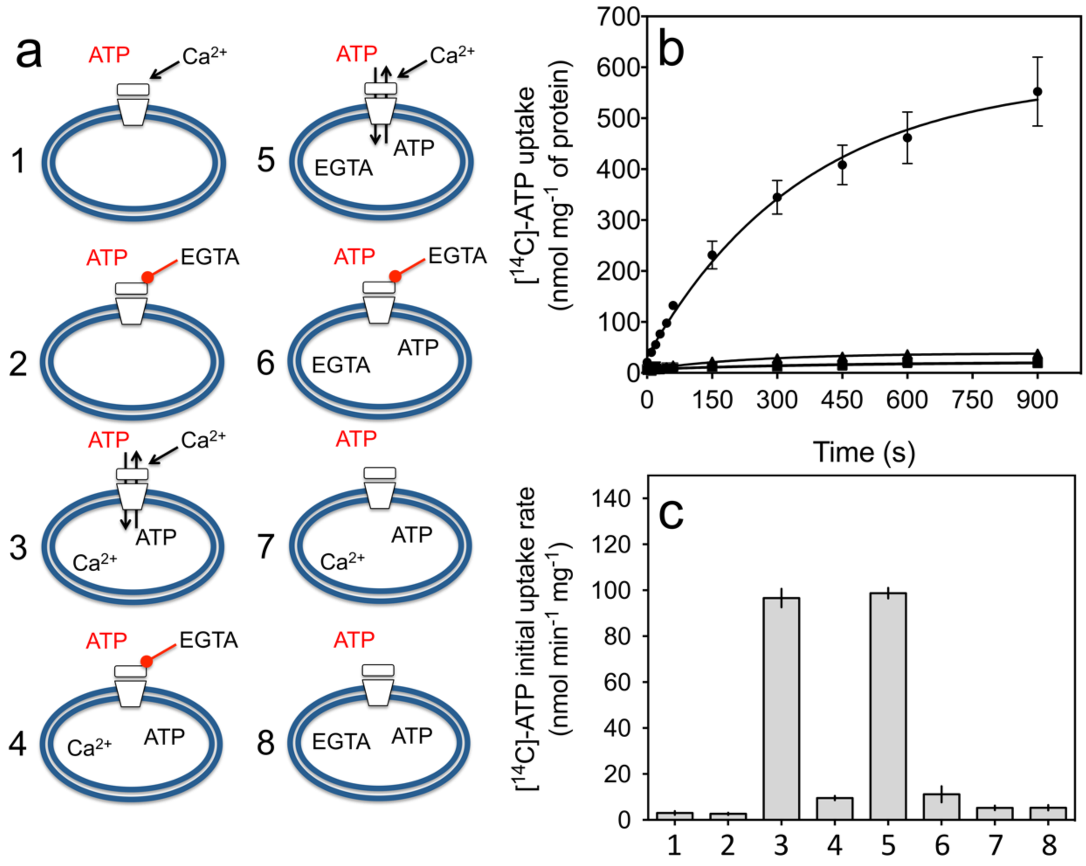

Supplementary Fig. 2 Regulation of transport and orientation of APC1 reconstituted into liposomes. a) Experimental setup of liposomes with different combinations of internal and external substrates and effectors are represented by schematic diagrams. The radiolabelled ATP is indicated in red and unlabelled substrate or effectors are black. b) Example [^{14}C]-ATP uptake into proteoliposomes loaded with 2.5 mM ATP and 1 mM calcium chloride (circles), 2.5 mM ATP and 1 mM EGTA (triangles) or no ATP and 1 mM EGTA (squares). All liposomes were loaded with 1 mM DTT and 20 mM tris-HCl pH 7.4. Uptake was initiated by the addition of 1.5 µM [^{14}C]-ATP to the outside of the liposome. The initial rates were calculated from the fit of the curves over the entire 15-minute time course. c) The uptake of 1.5 µM [^{14}C]-ATP into proteoliposomes was measured in the presence and absence of different combinations of unlabelled substrates and effectors, conditions are numbered as in panel A.

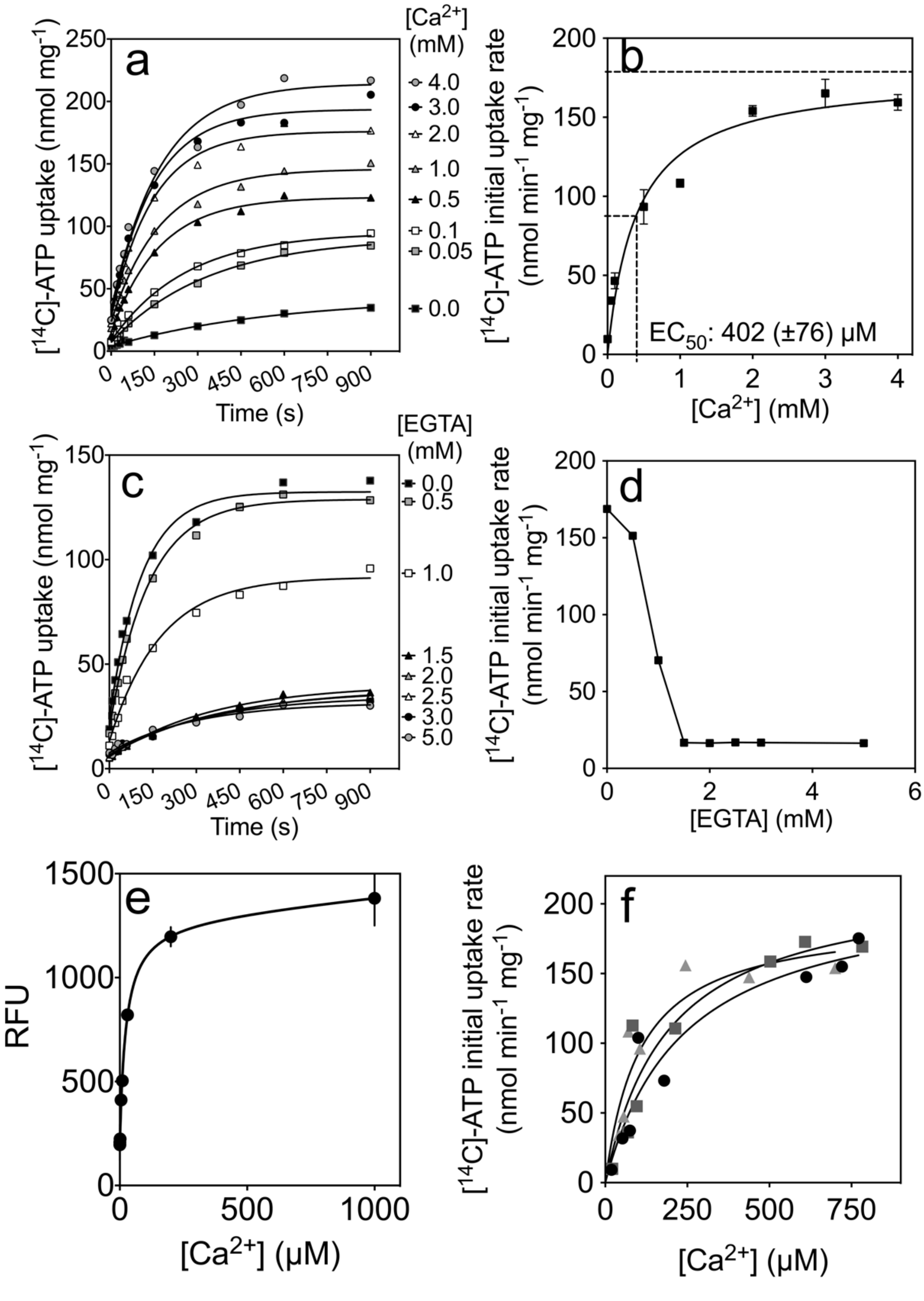

Supplementary Fig. 3 Kinetics of ATP transport at different calcium concentrations.

a) APC1 proteoliposomes loaded with 2.5 mM ATP, and uptake of 1.5 µM [^{14}C]-ATP stimulated by the addition of different concentrations of calcium (0, 0.05, 0.1, 0.5, 1, 2, 3 and 4 mM) on the outside. Error bars represent the standard deviation for measurements taken from three independent liposome preparations. b) The relationship between initial ATP uptake rate and the calcium concentration was plotted using the specific initial rates from panel A and fitted with a curve describing hyperbolic saturation kinetics. c) Uptake curves from APC1 proteoliposomes loaded with 2.5 mM ATP, in the presence of 1 mM calcium chloride and increasing concentrations of EGTA (0, 0.5, 1, 2, 2.5, 3 and 5 mM). Measurements were taken from a single liposome preparation. d) Specific initial rates of ATP uptake plotted as a function of EGTA concentration. Specific initial rates and the

EC_{50} value were estimated using least-squares curve fitting of the data by Prism (GraphPad). e) Standard curve of fluorescent signal (relative fluorescent units; RFU) from the low affinity calcium probe fluo-5N at 0.2, 1, 5, 10, 30, 200, 1000 µM calcium chloride. f) Data from panel b corrected to free-calcium concentration by interpolation of fluorescent signal against standard curve in panel e. Results from three independent liposome preps have been plotted individually and fitted with a curve describing hyperbolic saturation kinetics. The estimated V_{max} for ATP transport plotted with circles, squares and triangles was 214, 218 and 191 (mol min^{-1} mg^{-1}), respectively, and the estimated EC_{50} for calcium was 239, 191 and 107 (µM), respectively.

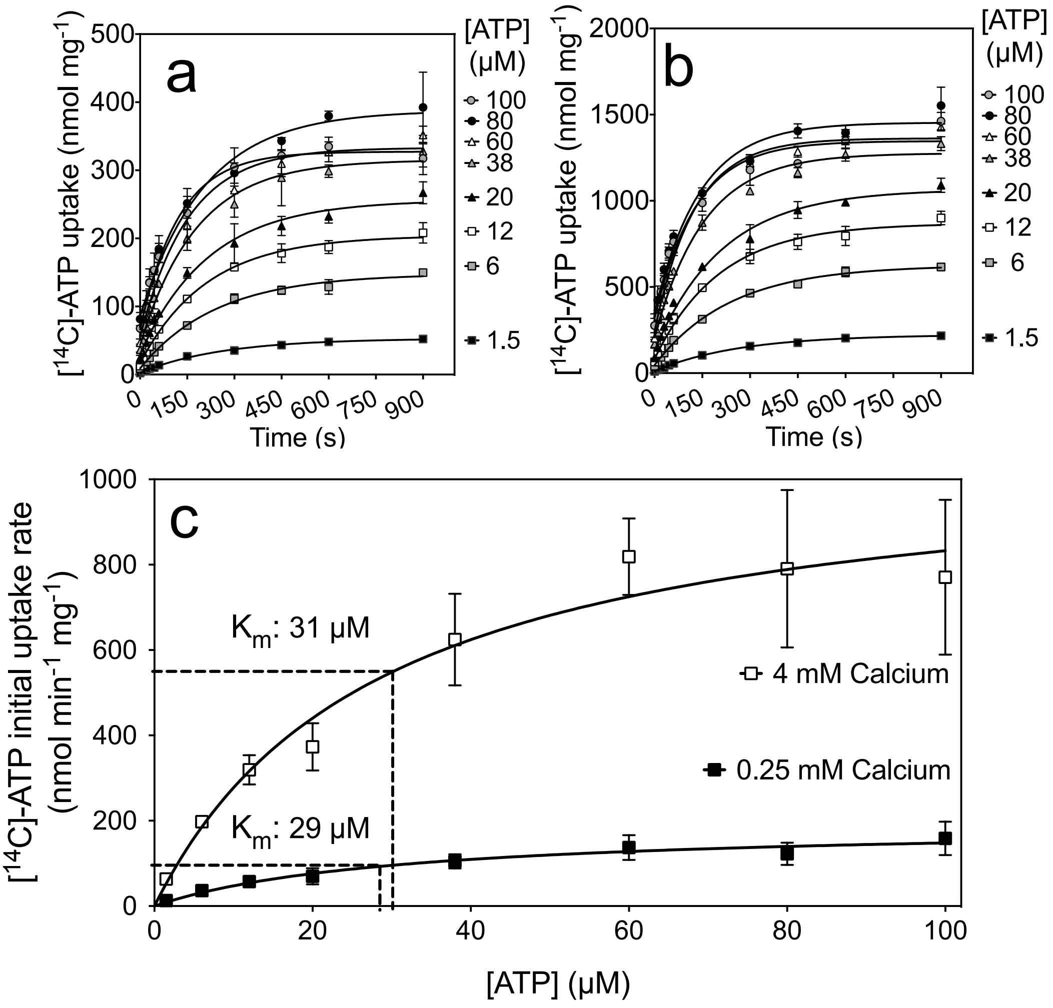

Supplementary Fig. 4 Kinetics of ATP transport at two calcium concentrations.

Uptake from APC1 proteoliposomes loaded with 2.5 mM ATP, with increasing concentrations of ATP (1.5, 6, 12, 20, 38, 60, 80 and 100 μM) added to the outside and in the presence of either a) 0.25 mM or b) 4 mM calcium chloride. All liposomes were loaded with 1 mM DTT and 20 mM tris-HCl pH 7.4. For ATP concentrations above 10 μM, [^{14}C]-ATP was mixed in a decreasing ratio with unlabelled ATP and the uptake rate was adjusted accordingly. c) The Michaelis-Menten relationship between initial ATP uptake rate and the ATP concentration was plotted for 0.25 mM (filled squares) and 4 mM (open squares) concentrations of external calcium using the specific initial rates from panel a and b, respectively. Error bars represent the standard deviation for measurements taken from three independent liposome preparations. Specific initial rates, K_m and V_{max} values were estimated using least-squares fitting of the data by Prism (GraphPad).

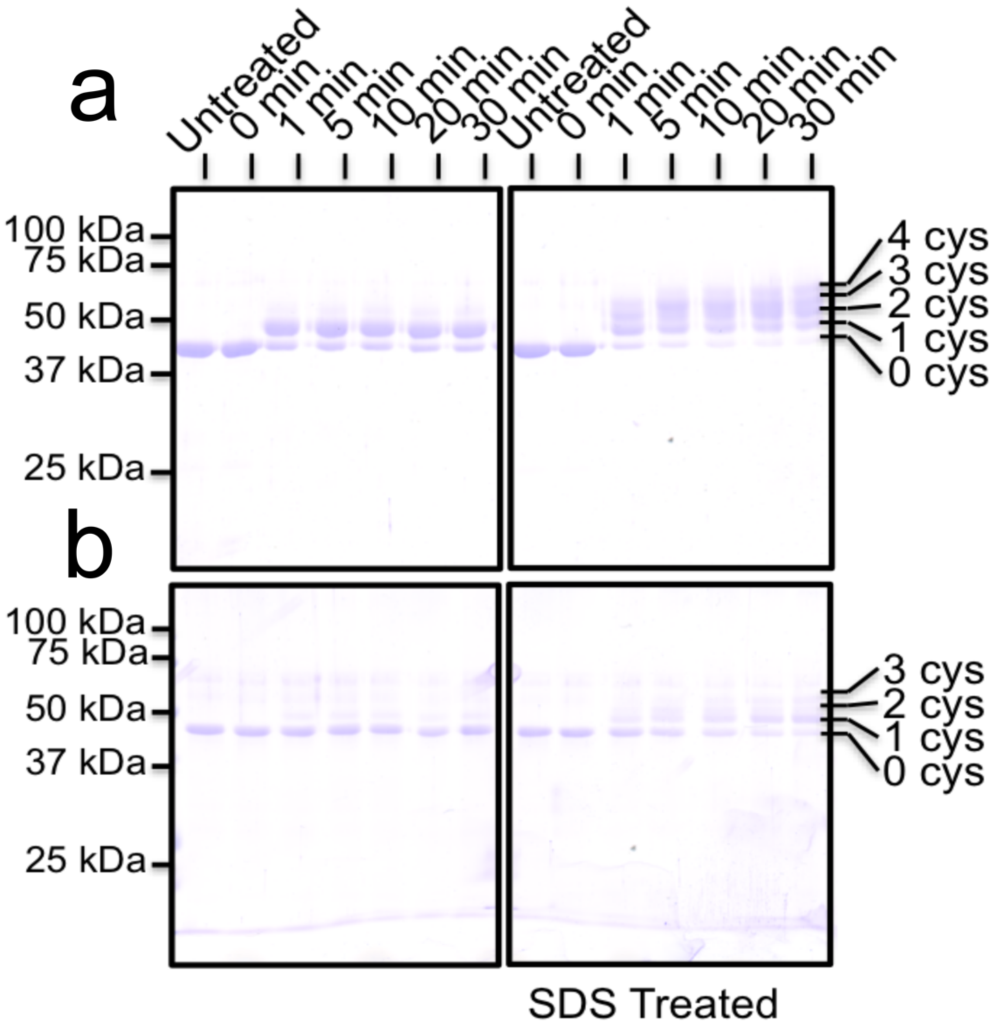

Supplementary Fig. 5 PEG-maleimide assay for the identification of surface exposed cysteines. SDS-PAGE gel analysis of PEG-maleimide 2K reacted A) wild-type APC1 and B) C15S mutant APC1, in the presence or absence of SDS. Samples were taken and quenched using DTT, before being mixed with SDS-PAGE gel loading buffer. Samples were run on an SDS-PAGE gel. For the PEG-maleimide reacted wild-type protein treated with SDS, 4 PEG modified cysteine residues can be observed, whereas in the C15S mutant, only 3 react as expected. In the non-SDS treated samples, one cysteine residue is modified, whereas in the C15S mutant, no cysteine residues are modified.

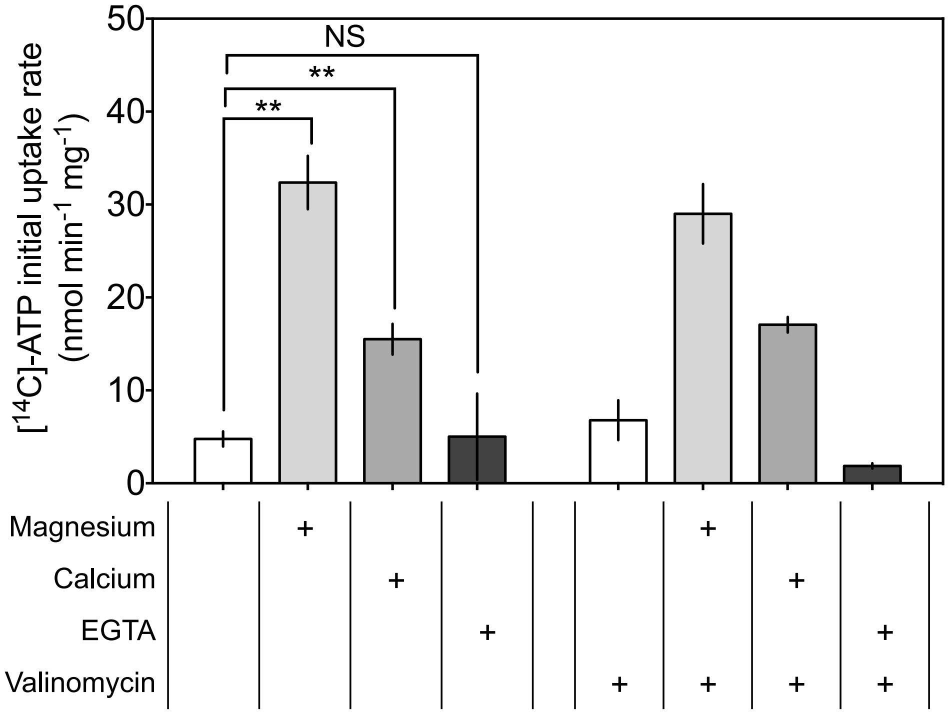

Supplementary Fig. 6 Effect of magnesium and calcium on ATP/ADP hetero-exchange by the carrier domain of APC1 alone.

The N-terminal truncation (Δ1-191_APC1) construct was reconstituted into liposomes and the amount of 1.5 µM [^{14}C]-ATP taken up, measured. All liposomes were loaded with 2.5 mM ADP, 1 mM DTT and 20 mM tris-HCl pH 7.4. Either 5 mM magnesium chloride, 5 mM calcium chloride or 5 mM EGTA was added to the outside of the liposome, and hetero-exchange between ATP and ADP was monitored over a 15-minute time course. The KCl concentration was 50 mM on the inside and outside of the liposomes and uptake in the presence and absence of valinomycin was tested in order to see what effect membrane potential had on the rate of the ATP/ADP exchange. Specific initial rates were estimated using least-squares fitting of the data by Prism (GraphPad). Statistical tests were calculated using unpaired, two-tailed Student's *t*-tests; NS $P > 0.05$; ** $P \leq 0.01$.

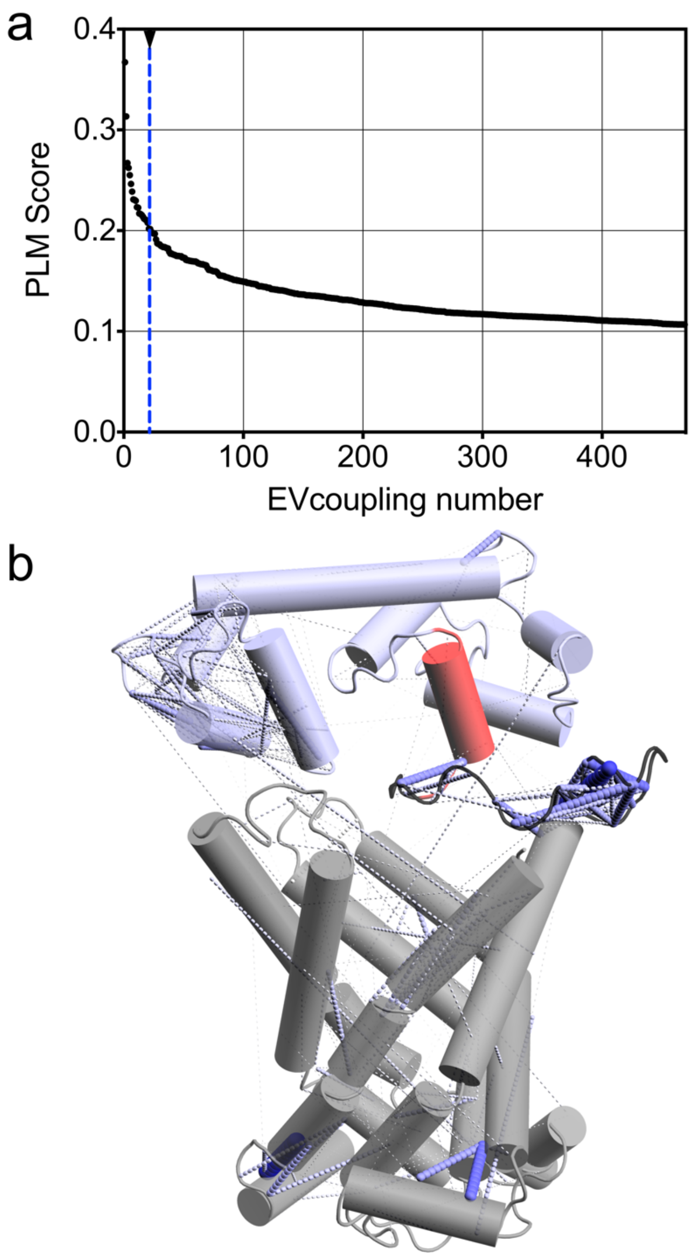

Supplementary Fig. 7 EVcoupling pairs provided by the EVfold server.

a) Scatter plot of EVcoupling pairs in rank order against PLM score. A distinct peak in PLM score is observed for EVcoupling pairs with a score above 0.2, indicated by a blue dashed line. b) Model of calcium-bound APC1 based on evolutionary coupling to map all 470 EVcouplings provided by the EVfold server. Blue dashed lines have been drawn between the Cβ atoms of the side chains of the EVcouple. The thickness and depth of blue colour correlates to the PLM score for each pair. In the model, the highest scoring couples are close to one another and interactions can be verified in the crystal structure of the regulatory domain, but as the score decreases, spuriously long distances are observed, suggesting only the top 20 highest scoring pairs can be trusted.

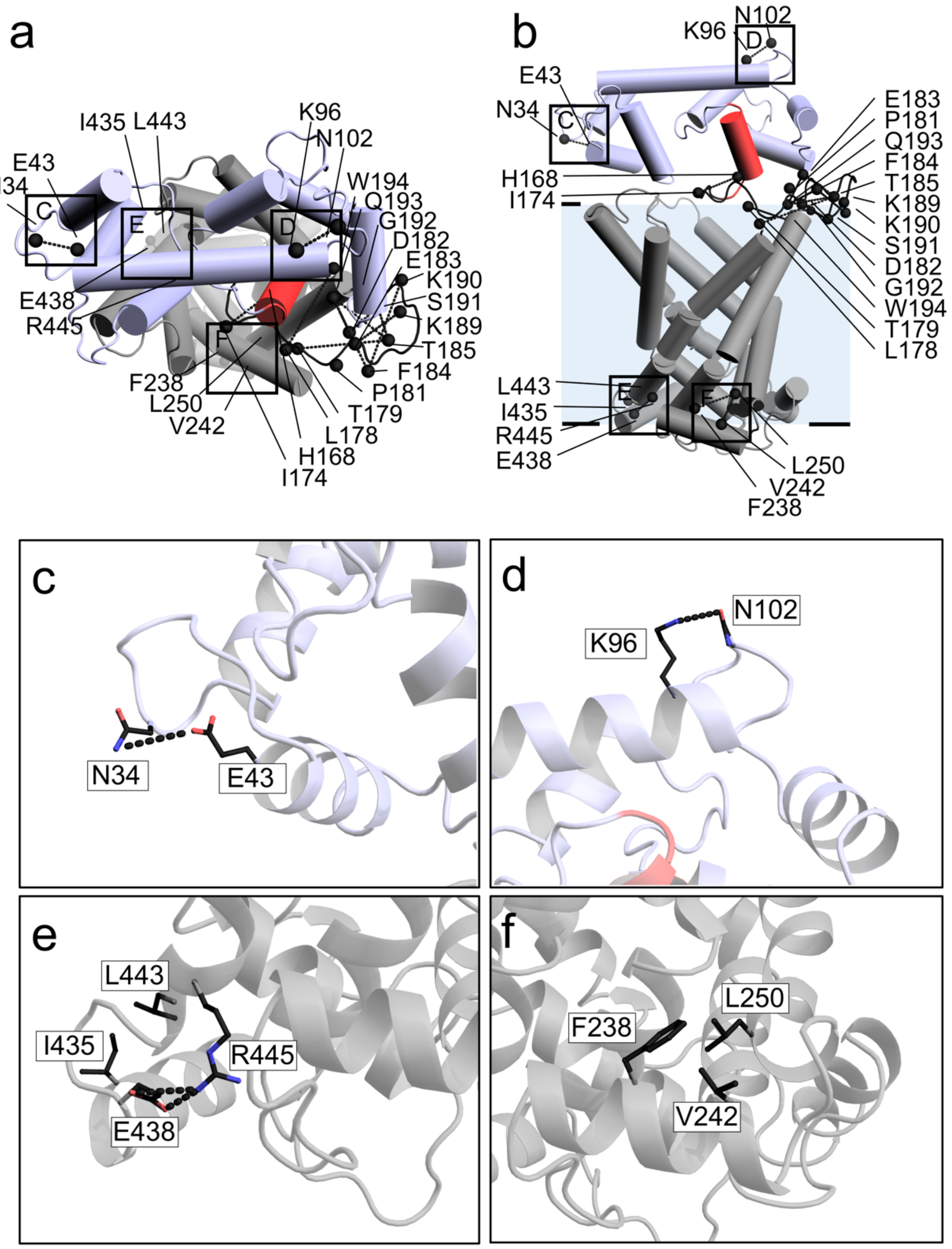

Supplementary Fig. 8 Model of calcium-bound APC1 based on evolutionary coupling showing refined set of EVcouplings.

a) View from the membrane and b) view from the cytoplasmic side of the APC1 model. Residues indicated to be involved in an evolutionary coupling pairs are represented as black spheres, and a dashed line has been drawn between the paired residues. The regulatory domain is coloured blue, the amphipathic α-helix red and the carrier domain grey. The relative positions of EV couplings pairs have been used to model the part of the protein with unknown structure and orient the regulatory domain relative to the carrier domain. Detailed views of EVcoupled pairs c) N34:E43, d) K96:N102, e) I435:L443, E438:R445, f) F238:L250 and V242:l250. Residues have been represented as sticks, and possible hydrogen bonding indicated by dashed lines.

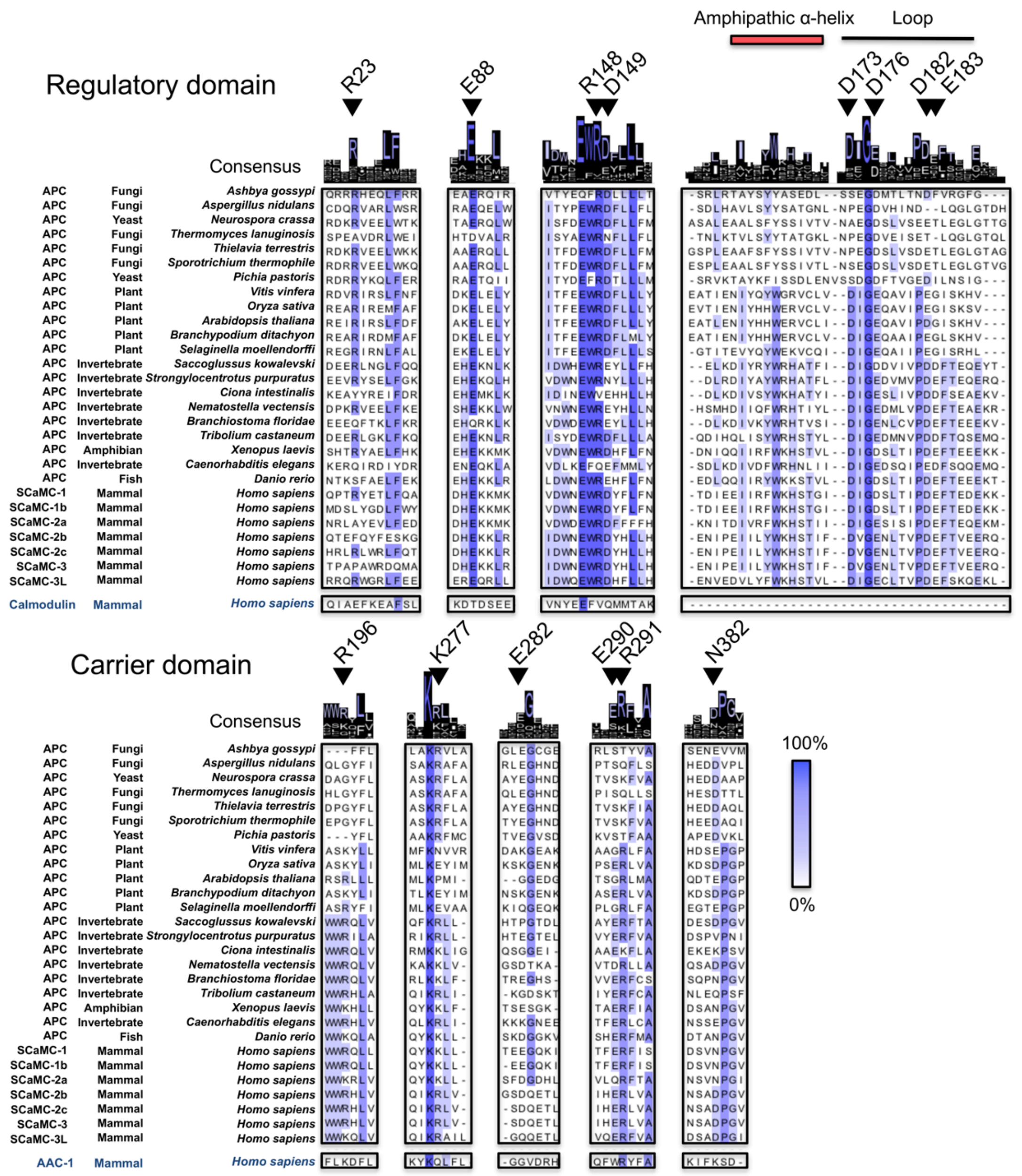

Supplementary Fig. 9 Sequence alignments of APC from mammal, invertebrates, plants and fungi to analyse conservation.

Alignments of APC orthologues from mammal, invertebrates, plants and fungi were prepared and the linker regions in the regulatory domain and the cytoplasmic loops of the carrier domain in APC orthologues are presented. In order to identify conserved residues that might mediate interactions between the regulatory domain and the carrier domain, APC sequences were compared with either human calmodulin or human ADP/ATP carrier isoform-1 (AAC-1), which highlight residues that are important for calcium binding in the regulatory domain or substrate transport in the carrier domain, respectively, and therefore not part of the interaction between the regulatory and carrier domains. Positions where charged residues are conserved, but not indicated to be involved with calcium binding or substrate transport and have been mutated to alanine in this study have been labelled according to the APC1 sequence. Residues are coloured according to the percentage of the residues in each column that agree with the consensus sequence as in the key. Only the residues that agree with the consensus residue for each column are coloured. Image of alignment prepared using Jalview[4].

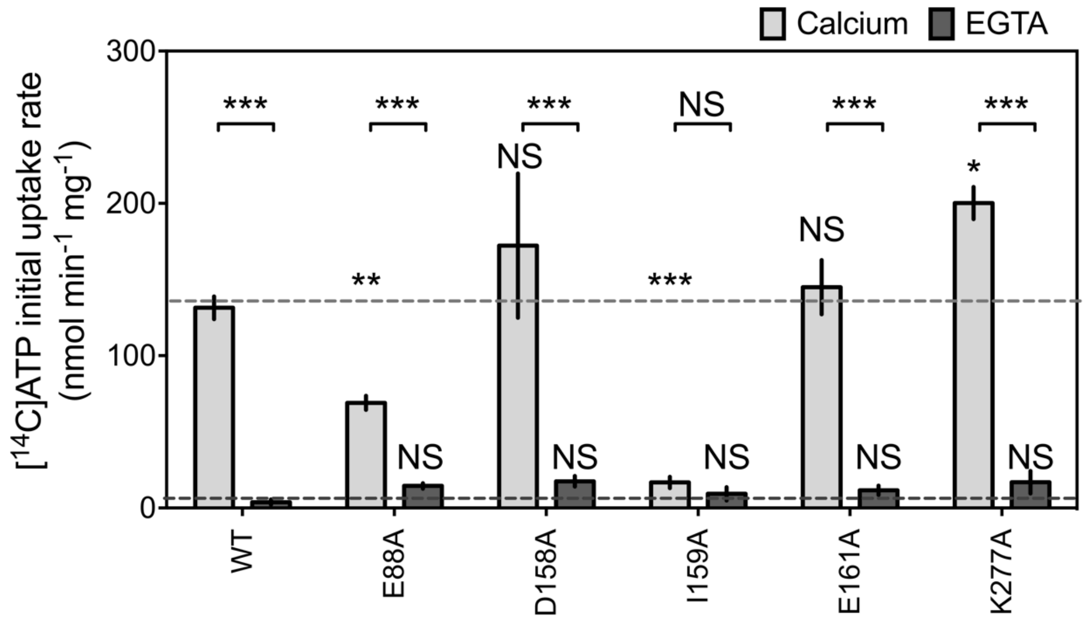

Supplementary Fig. 10 Effect of mutations on the uptake activity of APC1.

Specific initial uptake rate of 1.5 μM [^{14}C]ATP into proteoliposomes was measured. Proteoliposomes contained either wild-type or one of five mutant APC1. Each proteoliposome contained 2.5 mM ATP, 1 mM DTT and 20 mM tris-HCl pH 7.4, whereas the external buffer contained either 2.5 mM calcium chloride (light grey) or 2.5 mM EGTA (dark grey). Results were from three independent liposome preparations. Specific initial rates were fitted and significance values were calculated using unpaired, two-tailed Student's t-tests, where; NS $p > 0.05$; * $p \leq 0.05$; ** $p \leq 0.01$; *** $p \leq 0.001$. The significance between mutant and wild-type specific initial uptake rates in the presence (light grey dashed line) or absence of calcium (dark grey dashed line) are displayed above each bar. The significance between the specific initial uptake rate in the presence or absence of calcium is displayed above half-tick lines in each case. The final concentration of lipid in the assay was 0.5 mg mL^{-1}. The final protein concentration in each liposome sample was quantified by SDS-PAGE analysis, and compared to a standard curve of known protein concentration.

Supplementary Table 1. Top 20 scoring evolutionary couplings out of 470

First residue number	First residue	Second residue number	Second Residue	PLM score[a]
443	L	435	I	0.36717
193	Q	184	F	0.31335
189	K	183	E	0.26705
250	L	242	V	0.26232
192	G	184	F	0.25512
193	Q	185	T	0.24635
191	S	185	T	0.23865
174	I	168	H	0.23092
192	G	183	E	0.23047
185	T	179	T	0.22966
193	Q	183	E	0.22333
190	K	184	F	0.22276
194	W	178	L	0.21648
250	L	238	F	0.21551
102	N	96	K	0.21371
43	E	34	N	0.2118
192	G	181	P	0.21097
192	G	185	T	0.20787
445	R	438	E	0.20182
191	S	182	D	0.20159

[a] Pseudo-likelihood maximisation score, calculated by the EVfold server[5].

Supplementary Table 2. EVcoupling interactions used as restraints in HsAPC-1 model building

First residue number	First residue	First group	Second residue number	Second residue	Second group	Interaction distance in model
Residues from EVcouplings						
189	K	NZ	183	E	OE1	4.4
		NZ			OE2	3.9
193	Q	OE1	185	T	OG1	7.1
		NE2			OG1	6.5
191	S	OG	185	T	OG1	4.2
185	T	OG1	179	T	OG1	11.9
193	Q	NE2	183	E	OE1	4.4
		NE2			OE2	2.5
445	R	NH2	438	E	OE2	5.9
191	S	OG	182	D	OD1	6.4

Supplementary Movie 1. Proposed conformational changes involved in the *locking pin* mechanism of calcium regulation. The video highlights the proposed conformational change between the calcium-bound (active) and calcium-free (inactive) states of the ATP-Mg/Pi carrier. The calcium-bound regulatory domain in blue is based on PDB: 4ZCV. Residues of the blue and green clusters are displayed as colored spheres, representing the Cα atoms. Known and predicted interactions between residues in the different states have been indicated with dashed black lines. The calcium-free regulatory domain is based on a model in which all EF-hands of the regulatory domain are closed. The carrier domain in grey is a comparative model based on the bovine AAC crystal structure (PDB: 1OKC). The amphipathic α-helix is shown in red. The α-helices are represented as cylinders. The substrates ATP-Mg and phosphate, and activating calcium ions are in sphere representations.
